# Schizophrenia, gluten, and low-carbohydrate, ketogenic diets: a case report and review of the literature

**DOI:** 10.1186/1743-7075-6-10

**Published:** 2009-02-26

**Authors:** Bryan D Kraft, Eric C Westman

**Affiliations:** 1 Department of Medicine, Duke University Medical Center, DUMC Box # 31179, 2301 Erwin Road, Durham, NC 27710, USA; 2 Division of General Medicine, Department of Medicine, Duke University Medical Center, 4020 North Roxboro Street, Durham, NC 27704, USA

## Abstract

We report the unexpected resolution of longstanding schizophrenic symptoms after starting a low-carbohydrate, ketogenic diet. After a review of the literature, possible reasons for this include the metabolic consequences from the elimination of gluten from the diet, and the modulation of the disease of schizophrenia at the cellular level.

## Case report

C.D. is a 70 year-old Caucasian female with a diagnosis of schizophrenia since the age of seventeen. Her diagnosis was based on paranoia, disorganized speech, and hallucinations. She reported both auditory and visual hallucinations, including seeing skeletons and hearing voices that told her to hurt herself. According to her history, she has had these hallucinations on almost a daily basis since the age of seven. C.D. has also been hospitalized at least five times over the last six years for suicide attempts and increased psychotic symptoms. She has attempted to overdose on medications, cut herself, and ingest cleaning agents. Her most recent hospitalization was five months prior to initiating the low-carbohydrate diet. She has discussed both her suicidal ideations and her hallucinations with her psychiatrist who has tried to optimize her medication regimen in an effort to improve her symptoms, but this has been largely unsuccessful. Her prior anti-psychotic and mood-stabilizing medication regimen has included lithium 900 mg qhs, olanzapine (dose unknown), ziprasidone 40 mg bid, aripiprazole 30 mg qhs, lamotrigine 100 mg bid, and quetiapine 900 mg qhs. She is currently managed on risperidone 4 mg qhs.

C.D.'s other medical problems (and approximate year of diagnosis) included obesity (1950's), hypertension (1970's), depression (1940's), obstructive sleep apnea (2002), gastroesophageal reflux disease (2003), urinary incontinence (2002), glaucoma (1999), trochanteric bursitis (2004), peripheral neuropathy of unknown etiology (2006), and prior cholecystectomy (1978). Her current medications included atenolol 100 mg daily, furosemide 20 mg daily, trazodone 100 mg qhs, sertraline 100 mg daily, timolol eye drops 1 drop each eye bid, brimonidine eye drops 1 drop each eye bid, and vitamin E 400 IU every other day.

A typical day's diet consisted of the following: egg and cheese sandwich, diet soda, water, pimento cheese, barbequed pork, chicken salad, hamburger helper, macaroni and cheese, and potatoes. She rated her baseline fatigue as a "3" using a standardized questionnaire (0 = none, 4 = severe or frequent). Her body weight was 141.4 kilograms (BMI 52.6 kg/m^2^), sitting blood pressure (BP) was 130/72 mmHg, and pulse was 68 beats per minute. Physical examination showed an obese, mildly disheveled female with poor attention to hygiene, but was otherwise unremarkable. She was instructed how to follow a dietary regimen consisting of unlimited meats and eggs, 4 ounces of hard cheese, 2 cups of salad vegetables, and 1 cup of low-carbohydrate vegetables per day. This diet restricts carbohydrate intake to fewer than 20 grams per day [1].

She returned for a follow-up appointment 7 days after starting the low-carbohydrate diet. She was feeling well, and noted an increase in energy. She was seen again in clinic 19 days later. When asked how she was doing, she responded that she was no longer hearing voices or seeing skeletons. She first noticed this upon awakening about 8 days after starting the program. She had had no change in medication. The only change had been in her dietary intake which now consisted of beef, chicken, turkey, ham, fish, green beans, tomatoes, diet drinks, and water. She denied hunger. C.D. was very happy that she was no longer hearing voices, and believed that it made her calmer. Her body weight was 136.2 kilograms, sitting BP was 150/84 mmHg, and pulse was 76 beats per minute.

Over the course of 12 months, C.D. has continued the low-carbohydrate, ketogenic diet and has had no recurrence of her auditory or visual hallucinations. She has also continued to lose weight (body weight 131.4 kilograms) and experience improvements in her energy level. She acknowledged having 2–3 isolated episodes of dietary non-compliance that lasted several days, where she ate pasta, bread, and cakes around the winter holidays; however she had no recurrence of her hallucinations.

## Discussion

In this case study, the abrupt resolution of longstanding schizophrenic symptoms was observed after the initiation of a low-carbohydrate, ketogenic diet used for weight loss. Previously, Dohan observed a decrease in hospital admissions for schizophrenia in countries that had limited bread consumption during World War II, which suggested a possible relationship between bread and schizophrenia [2]. Dohan and colleagues also observed that overt schizophrenia was rare in remote tribal areas of several South Pacific islands where grains were rare, as compared to similar populations which had a higher prevalence of overt schizophrenia and grain consumption [3]. Additionally, some researchers have previously noted an association between schizophrenia and celiac disease, an immune-mediated enteropathy that is triggered by the ingestion of gluten-containing grains [4].

The treatment of schizophrenia today is largely pharmacological, but we found several treatments previously used or studied related to nutritional factors. There have been several small controlled studies in which a gluten-free diet showed promise in ameliorating schizophrenic symptoms [4]. In one such study, approximately 10% of schizophrenic patients had improvement in their symptoms by elimination of dietary gluten [5]. Another uncontrolled pilot study using a ketogenic diet (which is typically also a gluten-free diet because the consumption of gluten-containing bread and starch is eliminated) also suggested symptomatic improvement among patients with schizophrenia [6]. This study was motivated by the observation that patients with schizophrenia tended to eat more carbohydrates immediately before a psychotic episode. Additionally, low-carbohydrate, ketogenic diets have a long history for the treatment of refractory pediatric epilepsy [7,8] and recently have been studied as a treatment for obesity and cardiometabolic risk reduction [9,10]. The mechanism of action for the anti-epileptic effect may be related to an increase in GABA activity which leads to a general reduction in excitation [11]. Ketosis was not confirmed in C.D. after starting the low-carbohydrate, ketogenic diet. While checking serum or urinary ketones may have provided more information to C.D.'s current metabolic state, ketosis itself may be more effect than cause if the underlying process is indeed an immune-mediated reaction to gluten. Still, this is a limitation to this report.

The diagnosis of celiac disease is often difficult to make, but serologic tests are available to assist in the diagnosis. In C.D.'s case, an anti-gliadin IgG assay was performed and was 13 units (negative < 20 units). While the assay was negative, it was limited by the fact that it was performed 3 months after the initiation of the low carbohydrate diet and thus in the absence of an antigenic stimulus. Additionally, biopsy-proven celiac disease without serological evidence is a known clinical entity [12].

Dietary conditions other than gluten-sensitivity, such as vitamin deficiencies, have also been associated with psychosis and schizophrenia. For example, deficiencies in folate, vitamin C, and niacin have been suggested to worsen the symptoms of schizophrenia [13]. Furthermore, one study examining the nutritional content of a low carbohydrate diet found that while there was similar intake of other vitamins and minerals, the consumption of fiber and vitamin C was less in the low carbohydrate diet compared to a low fat diet [14]. In C.D.'s case, she reported consuming sources of vitamin C (tomatoes) in her diet history, and her prescribed diet allowed for her to consume other vitamin C rich foods as well (i.e. squash). Still, had she consumed less than the recommended intake of vitamin C, her symptoms should have worsened instead of improved. It is also unlikely that she would become overtly deficient in vitamin C after 8 days, which is when her symptoms changed. The same argument can be made for folate; because the majority of folic acid is found in fortified breads and grains, it is logical to assume then that initiating a gluten-free diet would have worsened her symptoms, and not improved them. Moreover, the previously studied doses of niacin (3 g/day) and methylfolate (15 mg/day) in patients with schizophrenia to achieve clinical improvement are far greater than what would be consumed in a typical low carbohydrate diet [15,16]. Finally, patients with schizophrenia have been shown to consume less fiber than the general U.S. population [17], but there is no data to suggest that altering the fiber content of a diet will change the symptoms of patients with schizophrenia.

## Conclusion

While more research is needed to confirm the association between gluten intake and schizophrenia and whether dietary change can ameliorate schizophrenic symptoms, health care providers could consider screening patients with schizophrenia for celiac disease and/or augment the medical regimen with a gluten-free or low-carbohydrate, ketogenic diet.

## Competing interests

The authors declare that they have no competing interests.

## Authors' contributions

EW conceived of the report, obtained the patient's history and physical exam and drafted the manuscript. BK participated in the patient's history and physical exam, and drafted the manuscript. All authors read and approved the final manuscript.

## Consent

Written informed consent was obtained from the patient for publication of this case report and accompanying images. A copy of the written consent is available for review by the Editor-in-Chief of this journal.
